# Enhanced Cyclically Stable Plasticity Model for Multiaxial Behaviour of Magnesium Alloy AZ31 under Low-Cycle Fatigue Conditions

**DOI:** 10.3390/ma17184659

**Published:** 2024-09-23

**Authors:** Aljaž Litrop, Jernej Klemenc, Marko Nagode, Domen Šeruga

**Affiliations:** Faculty of Mechanical Engineering, University of Ljubljana, Aškerčeva cesta 6, SI-1000 Ljubljana, Slovenia; aljaz.litrop@fs.uni-lj.si (A.L.); jernej.klemenc@fs.uni-lj.si (J.K.); marko.nagode@fs.uni-lj.si (M.N.)

**Keywords:** magnesium alloy, cyclic plasticity modelling, multiaxial loading, AZ31 sheet metal

## Abstract

Magnesium alloys, particularly AZ31, are promising materials for the modern automotive industry, offering significant weight savings and environmental benefits. This research focuses on the challenges associated with accurate modelling of multiaxial cyclic plasticity at small strains of AZ31 under low-cycle fatigue conditions. Current modelling approaches, including crystal plasticity and phenomenological plasticity, have been extensively explored. However, the existing models reach their limits when it comes to capturing the complexity of cyclic plasticity in magnesium alloys, especially under multiaxial loading conditions. To address this gap, a cyclically stable elastoplastic model is proposed that integrates elements from existing models with an enhanced algorithm for updating stresses and hardening parameters, using the hyperbolic tangent function to describe hardening and ensure a stabilised response with closed hysteresis loops for both uniaxial and multiaxial loading. The model is based on a von Mises yield surface and includes a kinematic hardening rule that promises a stable simulation of the response of AZ31 sheets under cyclic loading. Using experimental data from previous studies on AZ31 sheets, the proposed model is optimised and validated. The model shows promising capabilities in simulating the response of AZ31 sheet metal under different loading conditions. It has significant potential to improve the accuracy of fatigue simulations, especially in the context of automotive applications.

## 1. Introduction

Magnesium and magnesium alloys, which are primarily composed of magnesium with the addition of other elements such as aluminium, zinc, and manganese to enhance mechanical properties and corrosion resistance, have enormous potential in the search for new materials in modern industries such as the automotive industry [1,2]. The low density and good mechanical properties of magnesium offer enormous potential for weight savings, which can improve fuel consumption and reduce environmental impacts [3,4]. The design of a lightweight magnesium body structure shows significant weight reduction and better performance when compared to a design with more conservative metallic materials, such as steel and aluminium alloys [5]. There are currently two main technologies to produce magnesium components. Due to the very good castability compared to other cast metals, e.g., aluminium alloys, magnesium alloys are already present in a limited number of body and chassis components [6,7]. However, components manufactured using forming processes have better mechanical properties than casted components; in particular, a higher fatigue strength, which is crucial for the dynamic loads that are present during operation [8,9,10]. Alaneme and Okotete studied the developments in improving the plastic deformability of Mg and its alloys and concluded that new metallic systems based on Mg have to be developed in order to find solutions to its poor deformability at low temperatures [11]. Although magnesium sheets exhibit good mechanical behaviour, they still lack formability [7,12,13]. To overcome this disadvantage though, Gryguć et al. recently presented an improvement in the fatigue properties of the AZ31B alloy, by forging sheet metal at different temperatures and deformation rates [14].

Most of the technological limitations and advantageous mechanical properties of magnesium alloys are due to their hexagonal, close-packed (HCP) structure and the phenomenon of twinning, which manifests itself as an unusual behaviour during elastic–plastic deformation [15,16]. The main limitation of forming at low temperatures is the anisotropic mechanical properties caused by a strong basal structure during plastic deformation. These can be minimised and overcome by the use of additives to the alloys [17,18,19]. Grain orientation has a significant influence on the mechanical behaviour of magnesium alloy AZ31 [20,21]. The anisotropy of wrought magnesium alloys was studied in detail by Shi et al. [22] and Sadeghi et al. [23], where the influence of Sr on the improvement of mechanical properties was investigated in different directions. Park et al. investigated the effect of anisotropy on the low-cycle fatigue of AZ31 [24]. In fatigue terms, “low-cycle” refers to fatigue that occurs under conditions with a relatively small number of high-strain cycles, leading to significant plastic deformation and potential failure. Zhu et al. performed monotonic experiments in different material orientations with respect to the rolling direction, and torsion experiments on samples along the thickness direction [25]. These experiments were used to evaluate an elastic–plastic self-consistent model considering twinning and untwinning [25]. Guo et al. conducted experimental and numerical investigations on twinning behaviour under uniaxial tension [26]. Yang et al. showed that the activation of the deformation mechanism is correlated with the grain orientation and the loading direction [20]. The stability of twins in Mg alloys was also investigated by Liu et al., and it was concluded that twin structures have good thermal stability in a wide temperature range [27]. Studies have shown that AZ31 is already so improved that it can be formed even at room temperature [28].

Experimental results of the stress–strain behaviour of magnesium alloys in sheet metal form show the specific shape of the hysteresis loops, which differ under tensile and compressive loads [29]. The causes of this phenomenon are the interchangeable mechanisms of twinning and untwinning [30,31,32]. Experimental studies have shown that these asymmetries can be even more pronounced at elevated temperatures [33]. When multiaxial loads with different amplitudes occur, the hysteresis loops exhibit even more specific shapes and constitutive modelling becomes even more challenging [34,35,36]. Iftikhar and Khan studied subsequent yield loci evolution during proportional and non-proportional multiaxial loading [37,38]. On the other hand, in pure shear loading there is a much less effective twinning mechanism, and consequently, the cyclic stress–strain response is symmetric [39,40,41]. Shear loading can be achieved as torsional loading [42,43] or as in-plane loading for sheet metal [44,45]. Multiaxial loading is hence understood as tensile and/or compressive load combined with shear load, where the material is subjected to stress or strain in multiple directions simultaneously, leading to complex deformation behaviours. Xiong et al. experimentally investigated four strain-controlled fully reversed loading paths, including proportional and non-proportional axial-torsional loading [46]. Similarly, Albinmousa and Jahed experimentally observed non-proportional loading with 45° and 90° phase angle shifts between axial and torsional loading [47]. Both studies concluded that twinning mechanisms played an important role in deformation under multiaxial loading and that non-proportional loading has a slightly negative effect on fatigue life. Similarly, Anes et al. came to comparable conclusions by investigating the relationship between proportional and non-proportional fatigue damage in AZ31 magnesium alloys [48]. Yang et al. presented modified shear–tensile and shear–compressive specimens, usable to incite different shear and normal stress ratios under quasi-static loading [49].

Constitutive models for modelling the response of magnesium alloys must be able to describe the special phenomena that occur when loads above the yield stress are applied [50]. The complexity of the constitutive model is further increased when directional anisotropy and asymmetric responses to yield stress depending on the direction of loading are taken into account [51,52]. Tong et al. investigated the possible reduction in yield stress asymmetry in tension and compression using the equal channel angular pressing method [53]. According to this phenomenon, the yield surface has its own characteristics, which can be well described by the yield surface proposed by Plunkett et al. [54].

There are two well-known approaches for constitutive modelling of the cyclic behaviour of magnesium and its alloys. The first approach is based on crystal plasticity and the second follows a phenomenological description of continuum plasticity [50]. The crystal plasticity models focus on the single crystal scale but can be then extended to polycrystalline models and implemented in solution methods such as FEM [55] and self-consistent methods [56,57]. These types of models can describe different plastic deformation mechanisms observed at the macroscopic scale based on the crystalline microstructure of Mg alloys [58,59]. The main problem with crystal plasticity modelling of plastic deformation of Mg alloys is the difficulty in describing the initiation and evolution of the mechanisms of twinning and detwinning during cyclic loading [60], as well as the high computational cost when applied to the macroscopic scale. In general, however, crystal plasticity models simulate the cyclic plasticity of Mg alloys well [50]. Li et al. recently developed a constitutive model of crystal plasticity that accounts for dislocation slip, twinning, and detwinning, and can reproduce the multiaxial ratcheting of AZ31 alloys [61]. Likewise, Bong et al. presented a crystal plasticity constitutive model, which provides time-efficient and accurate simulations for magnesium alloys at elevated temperatures [62].

On the other hand, phenomenological constitutive represent the material as a homogeneous continuum, without consideration of specific deformation mechanisms [63]. Most currently applied phenomenological constitutive models use either a von Mises yield surface with kinematic and isotropic strain hardening or an advanced anisotropic yield surface in combination with isotropic strain hardening only [54,64,65]. For the description of cyclic plasticity of AZ31, Nguyen et al. [66] recently also used a phenomenological model using separate von Mises yield surfaces to describe each of the plasticity mechanisms (twinning, detwinning and slip) occurring during plastic deformation. Cyclic plasticity refers to a material’s behaviour under repeated loading and unloading cycles, where the material experiences irreversible deformation, while cyclic plasticity modelling involves the development of mathematical frameworks to predict this complex behaviour accurately under various loading conditions. A similar approach was then followed by Lee et al. for modelling the response during a tension–compression–tension load sequence [67]. Vigneshwarean and Benzerga [68] investigated a comparison between the computationally efficient two-surface plasticity model and the computationally intensive crystal plasticity model. Further research was presented by Noban et al. [69] using a von Mises yield surface and a modified form of the multi-term Armstrong–Frederick hardening rule to successfully simulate the proportional and non-proportional multiaxial cyclic plasticity of AZ31. Roostaei and Jahed went a step further and introduced a similar model for multiaxial cyclic plasticity using Ziegler’s generalised asymmetric/anisotropic kinematic hardening rule [70] which was successfully implemented in commercial FEM software. Another approach, which uses an anisotropic yield surface and a modified hardening rule, was presented by Yoon et al. [71]. It is based on the Cazacu–Barlat yield criterion [65], in conjunction with a non-associated plastic flow rule, thereby accounting for the effects of r-value anisotropy within the material. The yield surface introduced by Cazacu, Plunkett and Barlat [54] was used by Muhammad et al. [72] to describe the cyclic behaviour of AZ31 in the case of tension–tension and compression–tension–compression loading. The model with adaptive yield surfaces depending on the deformation mechanisms was presented by Lei et al., who also described the ratchetting behaviour of AZ31 [73]. The concept of distortional hardening to model the plastic behaviour of AZ31 was considered by Lee et al. to account for different temperatures during the simulation [74,75]. Constitutive models, such as those introduced by Yoon and Lei et al., were developed to accurately predict the anisotropic, cyclic, temperature-dependent, and ratcheting behaviours of materials like AZ31. Murugesan et al. presented a model which can describe the deformation behaviour of AZ31 magnesium alloy using hybrid artificial neural network-based models [76]. Constitutive modelling of uniaxial behaviour, oriented specifically into variable amplitude loading, was presented by Šolinc et al. using Prandtl–Ishlinskii operators [77,78], whilst Klemenc et al. [79] and Dallmeier et al. [80] focused on the simulation of the behaviour considering the memory rules under low-cycle fatigue conditions.

Although there exist constitutive models trying to describe the response of AZ31 under multiaxial cyclic loading, they were originally intended for cast materials and hence were calibrated against tension–compression and torsion experiments on round specimens. Although sheet metal products used in the automotive industry are also subjected to normal and shear loading, which induce in-plane stresses, these models are not capable of describing the AZ31 sheet metal-specific response. With equal importance, if the constitutive model is to be used for simulations of cyclic loading, it should consistently ensure closure of hysteresis loops for either uniaxial tension–compression, biaxial shear, or multiaxial loading. Pandey et al. [81] proposed a promising cyclic plasticity model for predicting multiaxial asynchronous responses using the Ohno–Wang kinematic hardening rule and Tanaka’s non-proportionality parameter for simulating the responses of steel materials, but this has not yet been calibrated and validated for metals with hexagonal close-packed crystal structures. Recently, Anes et al. proposed a model developed to predict the cyclic, multiaxial stress–strain behaviour of AZ31B-F magnesium alloys under multiaxial cyclic loading specifically developed for Abaqus incremental plasticity software, and not as a stand-alone constitutive model [82,83]. A weakness of the proposed constitutive model for predicting the cyclic multiaxial stress–strain behaviour under multiaxial cyclic loading is its inability to accurately capture the shear behaviour of the alloy under non-proportional loading conditions.

In this paper, a modified cyclic plasticity model is proposed, developed upon a von Mises yield surface combined with Ziegler’s generalised asymmetric/anisotropic kinematic hardening rule [70] and a multiaxial constitutive model originally developed for materials with Masing behaviour under variable loads [84]. The modified model has been specifically targeted to achieve a stabilised elastoplastic response of magnesium alloy AZ31 in a sheet metal form and consistent closure of stress–strain hysteresis loops under both uniaxial and multiaxial loads under low-cycle fatigue conditions. This has been the primary focus of the research. Particular attention has also been paid to the extension of the stress-updating algorithm [84] so that the modified model could provide robust simulations in all loading directions. The secondary focus of the research has been to investigate how to obtain a stable response and achieve the closure of hysteresis loops. Design of a new optimisation algorithm enabled the determination of the optimal values of the material’s parameters. Moreover, the modified model would have to become an integral part of the algorithm in order to be able to carry out the optimisation procedure. The third area of focus of the research embraced the verification of the model under tension–compression, shear, and multiaxial loading conditions for AZ31 sheet metal. The developed model and the accompanying optimisation algorithm provide an accurate tool for simulating the stress–strain response of HCP sheet metal components under cyclic loading.

## 2. Material and Methods

The cyclic plasticity model presented in this paper is optimized for AZ31 sheet metal, which typically consists of approximately 3% aluminium, 1% zinc, and 0.3% manganese, with the remainder being magnesium. To ensure accuracy, test data from previous research conducted on AZ31 sheet metal with this composition were utilized. The AZ31 alloy used in our study was commercially purchased as sheet metal with 3 mm thickness. For uniaxial tensile–compressive loading, experimental data obtained by Šolinc et al. [77] for different strain levels were used so that all three plastic mechanisms—twinning, detwinning, and slip—representing the asymmetry of plastic flow, could be observed. For the experimental data on shear loading, the special shear test fixtures presented by Litrop et al. [44] were used. The symmetrical stress–strain response during shear loading and the Masing behaviour could clearly be seen. The experimental data were determined by the authors for testing different shear stress levels using Digital Image Correlation (DIC) [85]. The experimental data for multiaxial cyclic loading were taken from the existing literature [86].

The modified cyclic plasticity model has been developed based on the constitutive model presented by Simo [87] and extended as presented in the research of Roostaei et al. [70]. This constitutive model was upgraded with a stress update algorithm, as described by Šeruga et al. [84]. The model has been coded in Python language and developed for a material point. In the future, after additional testing, it is planned to extend the model for implementation in the finite element method. Detailed descriptions of the enhanced plasticity model and the new optimisation algorithm for the determination of the material parameters can be found in the chapter on calculation.

## 3. Calculation

**Figure 1:**
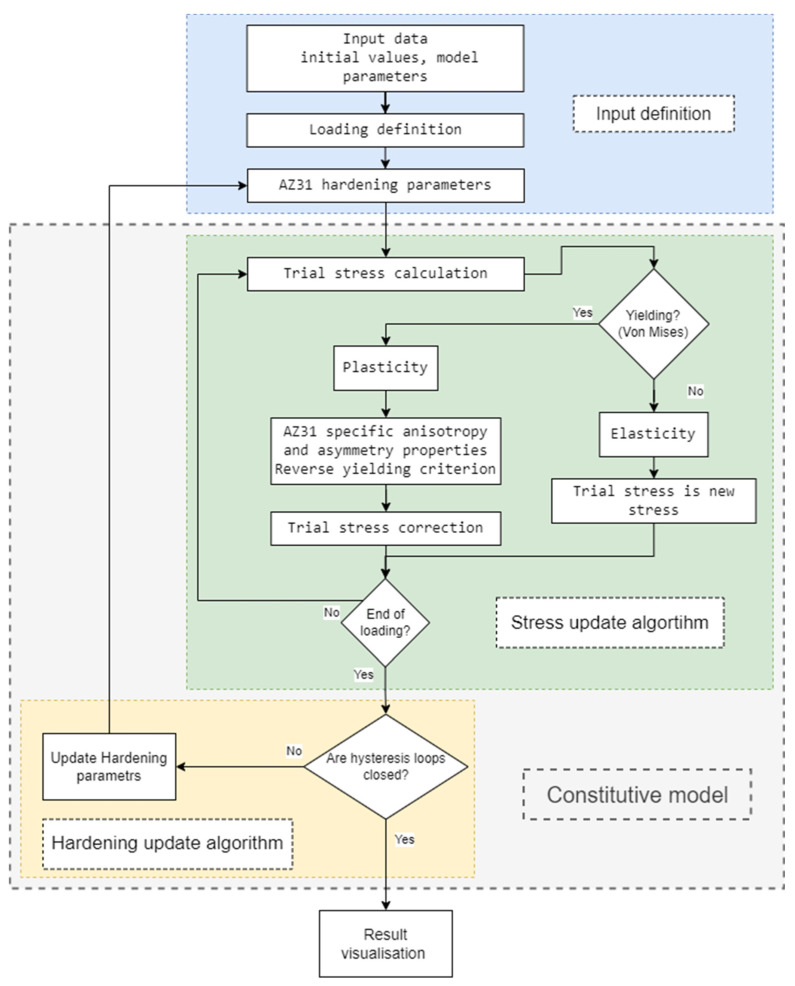
Constitutive model flowchart.

According to the specific mechanical behaviour at room temperature due to the mechanism of twinning and untwinning, the constitutive model should cover all peculiarities in modelling the cyclic plasticity response of AZ31 magnesium alloy. The constitutive model should consider all specific properties of AZ31, i.e., stress asymmetry and anisotropy, twinning and untwinning mechanisms, hardening behaviour, and the influence of multiaxial loading. The twinning and untwinning mechanisms are explicitly incorporated into the model through a phenomenological approach, which modifies the kinematic hardening rule to capture the tension–compression asymmetry characteristic of AZ31, employing a hyperbolic tangent function to precisely describe the transition between the twinning and untwinning mechanisms under varying load amplitudes. Furthermore, the enhanced model adheres to the principles of incremental plastic deformation calculation, utilizing the elastic predictor–plastic corrector scheme in conjunction with the forward Euler integration method to ensure stability and accuracy in the simulation of cyclic plasticity under complex loading conditions [70,84]. The flowchart of the proposed enhanced model is shown in Figure 1 and can be divided into four main areas.

### 3.1. Input Data Definition

In the first step, the input data, consisting of the material parameters, initial values, and the definition of the applied load, should be provided. In this part, the material parameters are the yield stress—$\sigma_{y}$; the modulus of elasticity—E; the Poisson ratio—ν; and the shear modulus—G. Model-specific parameters should also be specified. These include the size of the time increment required for the numerical implementation of the stress-updating algorithm. The initial values specify all the stresses and strains at the beginning of the simulation, which are usually all equal to zero if there is no prescribed history of the material. The next input is the definition of the load as a total strain increment $\Delta \tilde{\varepsilon }$, written in Voigt notation, which represents second-order tensor quantities (e.g., strains and stresses) and constitutive tensors (e.g., plastic modulus) as one- and two-dimensional arrays.

The input strain increment $\Delta \tilde{\varepsilon }$ can be specified as a triangular signal, sine, or another periodic signal independently for all components. The components can be in-phase or out-of-phase for tension–compression and shear deformation. The input strain increment component of the input strain vector $\Delta {\tilde{\varepsilon }}_{input,i+1}$ is defined for its components as a difference between the current step $\varepsilon_{input,k,i+1}$ and previous increment $\varepsilon_{input,k,i}$ of the defined strain loading shown in Equation (1).

(1) $$\Delta \varepsilon_{input,k,i+1}=\varepsilon_{input,k,i+1}-\varepsilon_{input,k,i};\;\;k=11,\;22,\;33,\;12,\;13,\;23$$

The last part in the input section of the proposed constitutive model is a specification of the hardening parameters used to compute the components of the hardening matrix $\left[H\right]$ used by the generalised Ziegler hardening rule [70]. The components of the hardening matrix $\left[H\right]$ essentially represent the material’s response under uniaxial cyclic loading conditions:

(2) $${\left[H\right]}_{i+1}=diag\left(H_{11,i+1},H_{22,i+1},H_{33,i+1},H_{12,i+1},H_{13,i+1},H_{23,i+1}\right)$$

In Equation (2), the components $H_{11},\;H_{22},\;\mathrm{and}\;H_{33}$ are used to describe uniaxial plastic behaviour under tensile and compressive loading and the shear hardening components $H_{12},\;H_{13},\;\;and\;H_{23},$ which are used to describe the shear plastic behaviour. The values of these components are determined from uniaxial tensile–compressive and shear testing. Specifically, they are defined as

(3) $$H_{k,i+1}=\left\{\begin{array}{c} \left[H_{0,L,U,i+1}+\nabla H_{L,k,i+1}\;\vartheta \left(N_{k,i+1}\right)+\nabla H_{U,k,i+1}\;\vartheta \left({-N}_{k,i+1}\right)\right]\;\left|m_{k,i+1}\right|;\;\;\;\;\;\;\;\;\;\;\;\;\;\;\;k=11,\;22,\;33\;\\ 2^{\left(1-n_{s}^{\prime }\right)}n_{s}^{\prime }K_{s}^{\prime }{\left(\frac{2^{\left(n_{s}^{\prime }-1\right)}\Sigma_{k,\;i+1}}{K_{s}^{\prime }}\right)}^{\frac{n_{s}^{\prime }-1}{n_{s}^{\prime }}}\;\left(\sqrt{2}\left|m_{k,i+1}\right|\right);\;\;\;\;\;\;\;\;\;\;\;\;\;\;\;\;\;\;\;\;\;\;\;\;\;\;\;\;\;\;\;\;\;\;\;\;\;\;\;\;\;\;\;k=12,\;13,\;23 \end{array}\right.$$

where $\nabla H_{L,U,k,i+1}$ is

(4) $$\nabla H_{L,U,k,i+1}=\frac{h_{L,U}}{2}\;\left[1+\tanh\left(\frac{\sigma_{k,i+1}-z_{L,U}}{d_{L,U\;}}\right)\right];\;\;k=11,\;22,\;33$$

In Equation (3), the parameter $H_{0,L,U}$ is the hardening at the flattened part of the unloading branch (compression—subscript “U”) and, on the other hand, the hardening at the inflection point on the loading branch (tension—subscript “L”) at the inflection point of the transition from the untwinning mechanism to the slip mechanism. $\vartheta \left(N_{K}\right)$ is a Heaviside function (Equation (5)) in respect to the outward normal to the yield surface $\tilde{N}$(Equation (6)) at the load point. Quantities ${\tilde{S}}^{Trial}$ and $\tilde{A}$ are the deviators of the stress tensor ${\tilde{\sigma }}_{i+1}^{Trial}$ and the back-stress tensor ${\tilde{\alpha }}_{i}$ and $\sigma_{Y}$ is the yield stress. The definition of the radial unit tensor m is shown as the difference between the stress and the back-stress divided by the Frobenius norm of the same difference as shown in Equation (7). The radial unit tensor m characterises the relative contributions of the uniaxial and shear loading components. The definition of back-stress tensor $\tilde{\alpha }$ is presented in the section titles ‘Stress Update Algorithm’.

(5) $$\vartheta \left(x\right)=\left\{\begin{array}{c} 1\;\;\;\;\;x\geq 0\\ 0\;\;\;\;\;x<0 \end{array}\right.\;$$

(6) $${\tilde{N}}_{i+1}=\frac{3}{2}\cdot \frac{{\tilde{S}}_{i+1}^{Trial}-{\tilde{A}}_{i}}{\sigma_{Y}}$$

(7) $${\tilde{m}}_{i+1}=\frac{{\tilde{\sigma }}_{i+1}^{Trial}-{\tilde{\alpha }}_{i}}{{\left\|{\tilde{\sigma }}_{i+1}^{Trial}-{\tilde{\alpha }}_{i}\right\|}_{F}}$$

In Equation (4) there are three dependent parameters for the loading (subscript “L”) and three for the unloading (subscript “U”). These parameters $h_{L,U}$, $z_{L,U},$ and $d_{L,U\;}$ are material parameters which must be defined separately for loading and unloading, and which depend on the magnitude of the load amplitude. These parameters are determined by applying a hyperbolic tangent function to describe the plastic hardening behaviour as a function of stress. The detailed procedure for fitting these parameters to the experimental data is given in Section 4.1. The shear hardening components in Equation (3) are dependent on the translated shear stress $\Sigma_{k,}$ which is in relation to the absolute value of the shear stress at the reversal point $\sigma_{k}^{R}$ and is calculated as

(8) $$\Sigma_{k,\;i+1}=\left|\left|\sigma_{k}^{R}\right|+Sgn\left(N_{k,\;i+1}\right){\cdot \sigma }_{k,\;i+1}\right|;\;\;k=12,\;13,\;23$$

A Ramberg–Osgood model, used for the description of shear hardening in Equation (3), has two parameters: the exponent of the cyclic shear hardening $n_{s}^{\prime }$ and the coefficient of the cyclic shear strength $K_{s}^{\prime }$. The parameters are independent of the direction and amplitude of the prescribed shear stress.

### 3.2. Stress Update Algorithm

After defining the required input data, the algorithm enters the main part—the stress update algorithm [87]. This algorithm calculates the new stress state for each time increment by using the elastic predictor—plastic corrector approach. The trial stress ${\tilde{\sigma }}^{Trial}$ as an elastic predictor is calculated considering Hooke’s law and assuming that the total strain increment $\Delta \tilde{\varepsilon }$ is fully elastic at the beginning of the time increment. The algorithm then proceeds with a verification of the yield condition by evaluating whether the equivalent von Mises stress exceeds the prescribed yield stress. If the yield condition is fulfilled, the trial stress is then corrected according to the hardening law (plastic corrector) and calculated for a current increment labelled i+1:

(9) $${\tilde{\sigma }}_{i+1}^{Trial}={\tilde{\sigma }}_{i}+\left[C\right]\cdot \Delta {\tilde{\varepsilon }}_{input,i+1}$$

In Equation (9), the stress ${\tilde{\sigma }}_{i}$ is the stress state from the previous increment. The initial value of the stress is zero for all components unless the material has a prescribed history. The matrix $\left[C\right]$ is the elastic stiffness matrix, defined as

(10) $$\left[C\right]=\left[\begin{array}{cccccc} 2G+\lambda & \lambda & \lambda & 0 & 0 & 0\\ \lambda & 2G+\lambda & \lambda & 0 & 0 & 0\\ \lambda & \lambda & 2G+\lambda & 0 & 0 & 0\\ 0 & 0 & 0 & G & 0 & 0\\ 0 & 0 & 0 & 0 & G & 0\\ 0 & 0 & 0 & 0 & 0 & G \end{array}\right],\;$$

where G is the shear modulus and λ is the second Lame’s constant. The trial equivalent stress is calculated using the von Mises yield criterion,

(11) $$\sigma_{VM,\;i+1}^{Trial}=\sqrt{\frac{3}{2}\cdot \left[{(S}_{i+1}^{Trial}-A_{i})∶\;{(S}_{i+1}^{Trial}-A_{i})\right]},$$

and is then compared with the prescribed yield stress $\sigma_{Y}$ using the trial variable called $f_{trial}$,

(12) $$f_{trial,\;i+1}=\sigma_{VM,\;i+1}^{Trial}-\sigma_{Y}<0$$

If the condition in Equation (12) is met, then an elastic increment is considered and the stress correction is not required. The new stress is the same as calculated in ${\tilde{\sigma }}_{i+1}^{Trial}$,

(13) $${\tilde{\sigma }}_{i+1}={\tilde{\sigma }}_{\;i+1}^{Trial}$$

All other variables that depend on plasticity are considered to be constant, e.g., the back-stress $\alpha_{k}$, the plastic strain $\varepsilon_{p,k}$ and the equivalent plastic strain increment Δp. If the condition in Equation (12) $f_{trial}<0$ is not fulfilled, plastic yielding sets in and the calculated increment must be corrected according to the prescribed hardening so that the stress is brought back to the yield surface. This procedure is referred to as the stress correction using the plastic corrector. To ensure phenomenologically correct calculation for HCP metals, this can be achieved by

(14) $$\sigma_{k,i+1}=\left\{\begin{array}{c} \alpha_{k,i+1}+\sigma_{Y}\cdot m_{\sigma ,k,i+1}+\sigma_{i+1}^{H}\;;\;\;\;\;\;\;\;\;\;\;\;\;\;\;\;\;\;\;\;\;k=11,\;22,\;33\\ \left[C\right]\cdot \left(\Delta \varepsilon_{input,k,i+1}-\Delta p_{i+1}\;N_{k,i+1}\right);\;\;\;\;\;\;\;\;\;\;k=12,\;13,\;23 \end{array}\right.$$

where hydrostatic stress $\sigma_{i+1}^{H}$ is calculated from the trial stress ${\tilde{\sigma }}_{i+1}^{Trial}$. The increment of the back-stress tensor $\Delta \tilde{\alpha }$ and the deviatoric part of the back-stress tensor can now be updated as

(15) $$\Delta {\tilde{\alpha }}_{i+1}=\left({\left[H\right]}_{i+1}\cdot {\tilde{m}}_{\sigma ,i+1}\right)\;\Delta p_{i+1}$$

and

(16) $$\Delta {\tilde{A}}_{i+1}=\left({\left[H\right]}_{i+1}\cdot {\tilde{m}}_{\sigma ,i+1}-\frac{1}{3}\tilde{I}\cdot \left({\tilde{I}}^{T}\cdot {\left[H\right]}_{i+1}\cdot {\tilde{m}}_{\sigma ,i+1}\right)\right)\;\Delta p_{i+1}$$

where Δp is the equivalent plastic strain increment and ${\tilde{m}}_{\sigma }$ is the tensor flow direction in Voigt notation. Δp is the scalar measure of the accumulated plastic strain increment during the current time step, representing the magnitude of plastic deformation that occurs when the yield condition is met. ${\tilde{m}}_{\sigma }$ represents the tensorial direction of plastic flow in the material, indicating the orientation in which plastic deformation occurs relative to the stress state. New values of the back-stress tensor ${\tilde{\alpha }}_{i+1}\;$and the deviatoric part of the back-stress tensor ${\tilde{A}}_{i+1}$ can now be calculated as

(17) $${\tilde{\alpha }}_{i+1}={\tilde{\alpha }}_{i}+\Delta {\tilde{\alpha }}_{i+1}\;$$

and

(18) $$A_{i+1}={\tilde{\alpha }}_{i}+\Delta {\tilde{A}}_{i+1}.$$

The forward Euler integration algorithm is employed as a closed-form expression to enhance computational efficiency by providing a straightforward and efficient method for updating the stress and strain increments in each time step [70]. Importantly however, flow direction, defined as [84]

(19) $${\tilde{m}}_{\sigma ,i+1}=\frac{{\tilde{\sigma }}_{i+1}^{Trial}-{\tilde{\alpha }}_{i}-\left(\tilde{I}\cdot \sigma_{i+1}^{H}\right)}{\sigma_{VM,\;i+1}^{Trial}}$$

must be used to ensure cyclically stable hysteresis modelling, resulting in the equivalent plastic strain increment Δp calculation equal to

(20) $$\Delta p_{i+1}=\frac{{\left({\tilde{S}}_{i+1}^{Trial}-{\tilde{A}}_{i}\right)}^{T}\cdot {\tilde{N}}_{i}-\sigma_{Y}\;}{{\left({\left[H\right]}_{i+1}\cdot {\tilde{m}}_{\sigma ,i+1}-\frac{1}{3}\tilde{I}\cdot \left({\tilde{I}}^{T}\cdot {\left[H\right]}_{i+1}\cdot {\tilde{m}}_{\sigma ,i+1}\right)\right)}^{T}\cdot {\tilde{N}}_{i}+3G}\;$$

Symbol $\tilde{I}$ stands for the identity tensor in Voigt notation whilst the matrix $\left[H\right]$ contains the plastic moduli or hardening for uniaxial tensile–compressive and shear loads for all directions [69]. The calculation of the components for uniaxial tensile–compressive and shear hardening parameters used in the matrix $\left[H\right]$ is provided in the Results section.

The main purpose of the improved stress update algorithm is to ensure the correct stress state according to the loading directions; i.e., uniaxial normal or shear loading, plane stress, or plane strain. The plastic corrector can, hence, successfully remove redundant stress components from the stress tensor in these cases. The proposed constitutive model also incorporates the reverse yielding criterion, which was first introduced by Lee et al. [88] and is essential for a cyclic plasticity model to distinguish the changes in the loading path. In this criterion, the angle between the two radial unit vectors in the neighbouring increments Λ is compared with the reference angle $\Lambda_{r}$ which is proposed as π/2 [70]. If the condition

(21) $$\Lambda_{i+1}=\cos^{-1}\left({\tilde{m}}_{i}^{T}\cdot {\tilde{m}}_{i+1}\right)\geq \Lambda_{r}\;\;$$

is fulfilled, then reverse yielding occurs, and the hardening parameters should be updated, otherwise forward yielding is considered. The criterion of reverse yielding is especially important where loads with different amplitudes induce nested cycles and cyclic memory rules must be applied.

### 3.3. Hardening Update Algorithm

**Figure 2:**
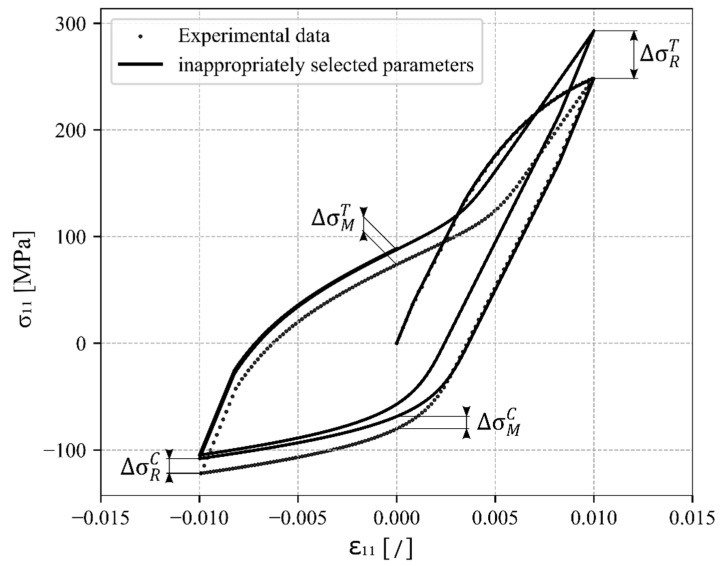
Hardening parameters update algorithm.

At this point the internal loop (iteration symbol—i) of the proposed constitutive model ends and the outer loop is entered (iteration symbol—j) which is responsible for ensuring that the hysteresis loops are stabilised and closed and that there is no drift at the reversal points in tensile or compressive directions. This is achieved by an additional optimisation algorithm which checks the difference between the calculated stress components of the neighbouring cycles in comparison to the permissible stress difference tolerance or in comparison to the difference to the experimental data for the first calibration, as shown in Figure 2.

Here, $\Delta {\tilde{\sigma }}_{R}^{T}$ defined as

(22) $$\Delta \sigma_{R,j}^{T}=\sigma_{R,i}^{T,N}-\sigma_{R,i}^{T,N-1}\leq \;\Delta \sigma_{R}^{T,Tol}$$

denotes the difference between two tensional reversal points of the neighbouring cycles N. The stress ${\tilde{\sigma }}_{R,i}^{T,N}$ belongs to the calculated ${\tilde{\sigma }}_{i}$ at the increment at the reversal point of the N-th cycle. The difference in the compression load is calculated in the same way,

(23) $$\Delta \sigma_{R,j}^{C}=\sigma_{R,i}^{C,N}-\sigma_{R,i}^{C,N-1}\leq \;\Delta \sigma_{R}^{C,Tol}.$$

If the conditions in Equations (22) and (23) are fulfilled, then the hardening parameters are considered to be set optimally and do not need further updating. If the criteria are not met, the hardening parameters must be further optimised so that the hysteresis loops are stabilised and closed. In this case the algorithm must recalculate the stress update for the entire input load.

To confirm that the hysteresis loops have the correct shape and stress values at reversal points when the strain load changes direction, additional stress difference tolerances $\Delta \sigma_{M}^{T}$ and $\Delta \sigma_{M}^{C}$ are specified:

(24) $$\Delta \sigma_{M,j}^{T}=\sigma_{M,i}^{T,N}-\sigma_{M,i}^{T,N-1}\leq \;\Delta \sigma_{M}^{T,Tol}$$

and

(25) $$\Delta \sigma_{M,j}^{C}=\sigma_{M,i}^{C,N}-\sigma_{M,i}^{C,N-1}\leq \;\Delta \sigma_{M}^{C,Tol}.$$

These two conditions are only used when the hardening parameters are still being calibrated with the experimental data. During the use of the constitutive model, they are no longer necessary.

The grid search method is used for updating the hardening parameters. The specific hardening parameters are increased or decreased until the difference conditions have been met. The algorithm for updating the hardening parameters updates $h_{L,U}$ and $z_{L,U}$ simultaneously at the end of each iteration of the outer loop. The updated hardening parameters (e.g., for tension) are calculated as follows:

(26) $$h_{L,\;j+1}=h_{L,\;j}+\Delta \sigma_{R,j}^{T}\cdot K_{h}$$

(27) $$z_{L,\;j+1}=z_{L,\;j}+\Delta \sigma_{R,j}^{T}\cdot K_{z}.$$

Convergence parameters $K_{h}$ and $K_{z}$ are used to define the sensitivity to parameter changes. The same convergence parameters are also used for compression (unloading). For corrections at reversal points, the convergence parameters can have the same or different values depending on the convergence rate. If these values are too high, the hardening parameters can be overcorrected, and then correction is required in the reverse direction in iteration j+1. This type of optimisation always ensures the closure of hysteresis loops and their correct shape. This refined approach integrated into the methodology is considered a crucial improvement over the original method, marking it as one of the more important novelties presented in this paper.

## 4. Results and Discussion

The proposed constitutive model for modelling of the cyclic stress–strain behaviour of magnesium alloys or other materials with hexagonal close-packed metals was tested and validated using the experimental data from AZ31 magnesium alloy under low-cycle fatigue conditions. First, the procedure for defining the hardening parameters for both the uniaxial normal and biaxial shear components is presented. Next, the validation of the constitutive model is demonstrated by predicting the response of AZ31 sheet metal under uniaxial tensile–compressive loading, pure shear loading, and as proportional loading.

### 4.1. Hardening Parameters Definition

Before using the proposed constitutive model in simulations, suitable functions of material parameters must be defined for the components of the hardening matrix $\left[H\right]$.

**Figure 3:**
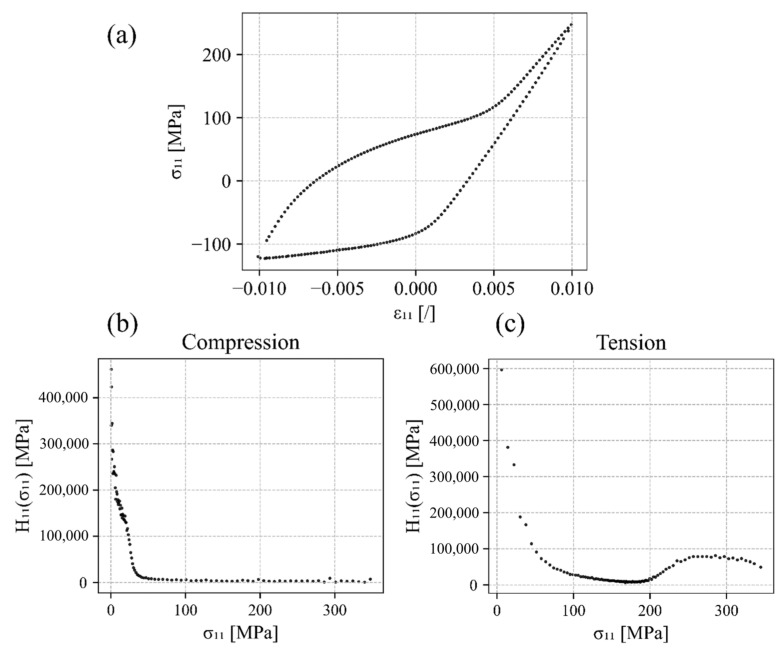
(**a**) Experimental results of the stabilised hysteresis loop for a uniaxial tensile–compressive load amplitude $\varepsilon_{a}=\pm 1\%$, (**b**) hardening component $H_{11}\left(\sigma_{11}\right)$ for the compression branch (downwards) of the hysteresis loop, and (**c**) hardening component $H_{11}\left(\sigma_{11}\right)$ for the tension branch (upwards).

First, the extraction of the hardening parameters $h_{L,U}$, $z_{L,U}$, $d_{L,U\;}$ and $H_{0\;L,U}$ from the experimental data under uniaxial loading/unloading is explained. The components of the hardening matrix $H_{k}$ where $k=\left(11,\;22,\;33\right)$ are extracted as the derivatives of the stress component $\sigma_{11}$ over the plastic strain component $\varepsilon_{p11}$. The plastic strain is obtained from the decomposition of the total strain into the elastic and plastic parts. Then, the function of plastic hardening component $H_{11}$ is defined as a function of the uniaxial tension–compression stress component $H_{k}\left(\sigma_{k}\right)$, which was previously calculated by Equation (3). The uniaxial stress component must then be translated with respect to the entire branch of the hysteresis loop so that the origin of the zero value starts at the reversal point and it only has positive (absolute) values. Figure 3a shows the experimental data of Šolinc et al. [77] for strain amplitude ± 1%, whilst Figure 3b,c are plots of $H_{k}\left(\sigma_{k}\right)$ extracted from the experimental data.

A hyperbolic tangent function is used to describe the parameters, since it can conveniently outline the S-shaped branches of the hysteresis loop. When adjusting the hardening parameters for the compressive branch (downwards) of the hysteresis loop, four parameters are used. The parameter $H_{0U}$ can be determined as the value of $H_{k}\left(\sigma_{k}\right)$ when it no longer changes significantly in its gradient (the curve is flattened). The other parameters $h_{U}$, $z_{U}$ and $d_{U\;}$ are determined using the non-linear least squares method between the experimental data and the hyperbolic tangent function.

**Figure 4:**
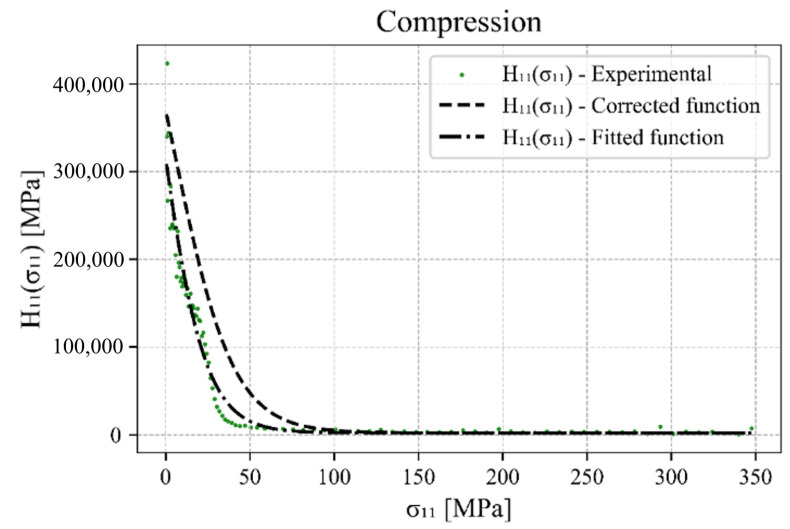
$H_{11}\left(\sigma_{11}\right)$ for the compression branch of the hysteresis loop with adapted hyperbolic tangent function.

**Figure 5:**
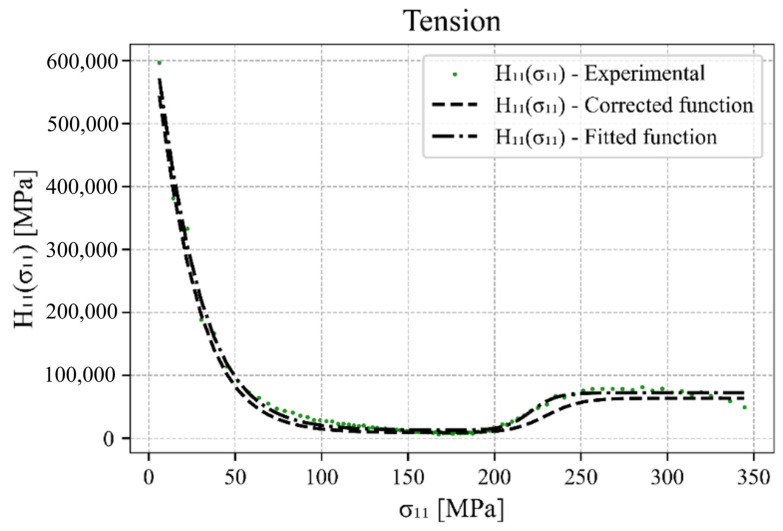
$H_{11}\left(\sigma_{11}\right)$ for the tension branch of the hysteresis loop with adapted hyperbolic tangent function.

A similar procedure is followed for the tensile branch (upwards) of the hysteresis loop, but with one significant difference. Due to the changing deformation mechanisms, which range from detwinning to slipping and then twinning again at higher strain, there is a specific S-shaped tensile branch of the hysteresis loop for which two sets of material parameters are required. The material parameters $h_{L1}$, $z_{L1}$ and $d_{L1\;}$ for untwinning and $h_{L2}$, $z_{L2}$ and $d_{L2\;}$ for twinning. The plastic hardening in the centre part or at the inflection point of the tensile branch of the hysteresis loop is described by $H_{0L}$, which in turn is conveniently identified by the value of $H_{k}\left(\sigma_{k}\right)$ once it no longer changes significantly in its gradient and is flattened. The other hardening parameters are again determined using the non-linear least squares method between the experimental data and the hyperbolic tangent function. The fitted function and the function corrected by the hardening parameters update algorithm for the compressive loading are presented in Figure 4. The same functions for the tensile loading can be found in Figure 5.

**Figure 6:**
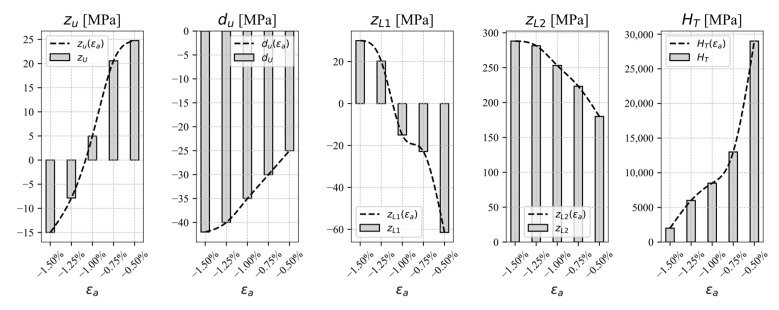
Functions of hardening parameters in dependency of the strain amplitude.

Since the size and the shape of the hysteresis loops depends on the applied load amplitude, the hardening parameters can be defined as functions of the load amplitude. It is recommended to calibrate the parameters for at least five strain amplitudes in steps of 0.25%. The interpolation function can then be used to describe the parameters between the experimental curves. Here, a polynomial function has been used for the interpolation, but it should be noted that it was limited to the range of strain amplitudes used in the calibration. The interpolations for the hardening parameters of the observed experimental data are shown in Figure 6. Table 1 shows the cyclic uniaxial tensile–compressive and pure shear hardening parameters from experimental results for AZ31. The parameters, which are functions of the strain amplitude, are presented as piecewise cubic interpolation functions

(28) $$X\left(\varepsilon_{a}\right)=\left\{A_{1,m}\;\varepsilon_{a}^{3}+A_{2,m}\;\varepsilon_{a}^{2}+A_{3,m}\;\varepsilon_{a}+A_{4,m},\;\;\;if\;\varepsilon_{a}\in m\;\right.\;$$

where m is

(29) $$m=\left[\left[-0.015,-0.0125\right],\;\left(-0.0125,-0.01\right],\;\left(-0.01,-0.0075\right],\;\left(-0.0075,-0.005\right]\right]$$

Hardening parameters $z_{U}\left(\varepsilon_{a}\right)$, $d_{U}\left(\varepsilon_{a}\right)$, $z_{L1}\left(\varepsilon_{a}\right),\;z_{L2}\left(\varepsilon_{a}\right),\;H_{0L}\left(\varepsilon_{a}\right)$ as functions of strain amplitude are presented as piecewise cubic interpolation functions:

(30) $$z_{U}\left(\varepsilon_{a}\right)=\left\{\begin{array}{c} \begin{array}{c} 7.17\cdot 10^{7}\varepsilon_{a}^{3}+3.23\cdot 10^{6}\varepsilon_{a}^{2}+5.08\cdot 10^{4}\varepsilon_{a}+2.63\cdot 10^{2},\;if\;\varepsilon_{a}\in \left[-0.015,-0.0125\right]\;\\ 4.60\cdot 10^{6}\varepsilon_{a}^{3}+7.11\cdot 10^{5}\varepsilon_{a}^{2}+1.93\cdot 10^{4}\varepsilon_{a}+1.32\cdot 10^{2},\;if\;\varepsilon_{a}\in \left(-0.0125,-0.01\right] \end{array}\\ \begin{array}{c} -2.77\cdot 10^{8}\varepsilon_{a}^{3}-7.75\cdot 10^{6}\varepsilon_{a}^{2}-6.53\cdot 10^{4}\varepsilon_{a}-1.50\cdot 10^{2},\;if\;\varepsilon_{a}\in \left(-0.01,-0.0075\right]\\ 2.01\cdot 10^{8}\varepsilon_{a}^{3}+3.02\cdot 10^{6}\varepsilon_{a}^{2}+1.55\cdot 10^{4}\varepsilon_{a}+5.20\cdot 10^{1},\;\;if\;\varepsilon_{a}\in \left(-0.0075,-0.005\right] \end{array} \end{array}\right.\;$$

(31) $$d_{U}\left(\varepsilon_{a}\right)=\left\{\begin{array}{c} \begin{array}{c} 5.14\cdot 10^{7}\varepsilon_{a}^{3}+2.31\cdot 10^{6}\varepsilon_{a}^{2}+3.52\cdot 10^{4}\varepsilon_{a}+1.34\cdot 10^{2},\;if\;\varepsilon_{a}\in \left[-0.015,-0.0125\right]\;\\ -6.51\cdot 10^{7}\varepsilon_{a}^{3}-2.05\cdot 10^{6}\varepsilon_{a}^{2}-1.94\cdot 10^{4}\varepsilon_{a}-8.89\cdot 10^{1},\;if\;\varepsilon_{a}\in \left(-0.0125,-0.01\right] \end{array}\\ \begin{array}{c} 1.71\cdot 10^{7}\varepsilon_{a}^{3}+4.11\cdot 10^{5}\varepsilon_{a}^{2}+5.23\cdot 10^{3}\varepsilon_{a}-6.64,\;if\;\varepsilon_{a}\in \left(-0.01,-0.0075\right]\\ -3.42\cdot 10^{6}\varepsilon_{a}^{3}-5.14\varepsilon_{a}^{4}+1.76\cdot 10^{3}\varepsilon_{a}-1.53\cdot 10^{1},\;\;if\;\varepsilon_{a}\in \left(-0.0075,-0.005\right] \end{array} \end{array}\right.\;$$

(32) $$z_{L1}\left(\varepsilon_{a}\right)=\left\{\begin{array}{c} \begin{array}{c} -5.95\cdot 10^{8}\varepsilon_{a}^{3}-2.68\cdot 10^{7}\varepsilon_{a}^{2}-4.02\cdot 10^{5}\varepsilon_{a}-1.98\cdot 10^{3},\;if\;\varepsilon_{a}\in \left[-0.015,-0.0125\right]\;\\ 1.35\cdot 10^{9}\varepsilon_{a}^{3}+4.62\cdot 10^{7}\varepsilon_{a}^{2}+5.11\cdot 10^{5}\varepsilon_{a}+1.82\cdot 10^{3},\;if\;\varepsilon_{a}\in \left(-0.0125,-0.01\right] \end{array}\\ \begin{array}{c} -1.43\cdot 10^{9}\varepsilon_{a}^{3}-3.74\cdot 10^{7}\varepsilon_{a}^{2}-3.26\cdot 10^{5}\varepsilon_{a}-9.68\cdot 10^{2},\;if\;\varepsilon_{a}\in \left(-0.01,-0.0075\right]\\ 6.81\cdot 10^{8}\varepsilon_{a}^{3}+1.02\cdot 10^{7}\varepsilon_{a}^{2}+3.13\cdot 10^{4}\varepsilon_{a}-7.49\cdot 10^{1},\;\;if\;\varepsilon_{a}\in \left(-0.0075,-0.005\right] \end{array} \end{array}\right.\;$$

(33) $$z_{L2}\left(\varepsilon_{a}\right)=\left\{\begin{array}{c} \begin{array}{c} -3.76\cdot 10^{8}\varepsilon_{a}^{3}-1.69\cdot 10^{7}\varepsilon_{a}^{2}-2.54\cdot 10^{5}\varepsilon_{a}-9.88\cdot 10^{2},\;if\;\varepsilon_{a}\in \left[-0.015,-0.0125\right]\;\\ 5.06\cdot 10^{8}\varepsilon_{a}^{3}+1.61\cdot 10^{7}\varepsilon_{a}^{2}+1.59\cdot 10^{5}\varepsilon_{a}+7.36\cdot 10^{2},\;if\;\varepsilon_{a}\in \left(-0.0125,-0.01\right] \end{array}\\ \begin{array}{c} -3.76\cdot 10^{8}\varepsilon_{a}^{3}-1.03\cdot 10^{7}\varepsilon_{a}^{2}-1.05\cdot 10^{5}\varepsilon_{a}-1.46\cdot 10^{2},\;if\;\varepsilon_{a}\in \left(-0.01,-0.0075\right]\\ 2.47\cdot 10^{8}\varepsilon_{a}^{3}+3.71\cdot 10^{6}\varepsilon_{a}^{2}-3.26\cdot 10^{2}\varepsilon_{a}+1.16\cdot 10^{2},\;\;if\;\varepsilon_{a}\in \left(-0.0075,-0.005\right] \end{array} \end{array}\right.\;$$

(34) $$H_{0L}\left(\varepsilon_{a}\right)=\left\{\begin{array}{c} \begin{array}{c} -2.17\cdot 10^{10}\varepsilon_{a}^{3}-9.77\cdot 10^{8}\varepsilon_{a}^{2}-1.29\cdot 10^{7}\varepsilon_{a}-4.52\cdot 10^{4},\;if\;\varepsilon_{a}\in \left[-0.015,-0.0125\right]\;\\ 1.25\cdot 10^{10}\varepsilon_{a}^{3}+3.08\cdot 10^{8}\varepsilon_{a}^{2}+3.15\cdot 10^{6}\varepsilon_{a}+2.17\cdot 10^{4},\;if\;\varepsilon_{a}\in \left(-0.0125,-0.01\right] \end{array}\\ \begin{array}{c} 1.95\cdot 10^{11}\varepsilon_{a}^{3}+5.79\cdot 10^{9}\varepsilon_{a}^{2}+5.80\cdot 10^{7}\varepsilon_{a}+2.04\cdot 10^{5},\;if\;\varepsilon_{a}\in \left(-0.01,-0.0075\right]\\ -1.86\cdot 10^{11}\varepsilon_{a}^{3}-2.79\cdot 10^{9}\varepsilon_{a}^{2}-6.40\cdot 10^{6}\varepsilon_{a}+4.35\cdot 10^{4},\;\;if\;\varepsilon_{a}\in \left(-0.0075,-0.005\right] \end{array} \end{array}\right.\;$$

For the simulation of the stress–strain response, the static or the stabilised cyclic curve must also be defined. The hardening parameters of the hysteresis loops cannot be used in this case, since the curves are different in their sizes and shapes. Therefore, the static hardening functions for uniaxial normal and biaxial shear loadings are defined as follows. These function parameters are determined by calibrating the function to the experimental data of uniaxial plastic hardening as a function of stress.

(35) $$H_{static,11}\left(\sigma_{11}\right)=A_{11}\cdot {\left(\sigma_{11}-\sigma_{0,11}\right)}^{B_{11}}+C_{11}\;\;$$

(36) $$H_{static,12}\left(\sigma_{12}\right)=A_{12}\cdot {\left(\sigma_{12}-\sigma_{0,12}\right)}^{B_{12}}$$

The importance of the correct definitions of the hardening parameters is enormous, because the quality of the simulation depends on the exact definitions of the parameter values. If the values of the parameters are not accurately defined, there is a possibility that the reversal points of the hysteresis loops will drift, which looks like ratchetting. If the parameters are not correct, the twinning will occur too early or too late depending on the current load. For this reason, it is important to use an algorithm to update the hardening parameters that eliminates the drift of the reversal points. This approach is a principal contribution to improving the accuracy and predictability of simulations for AZ31. It represents a central objective of this paper, aiming to enhance the stability and reliability of the simulation outcomes.

### 4.2. Uniaxial Tensile–Compressive and Pure Shear Loading

**Figure 7:**
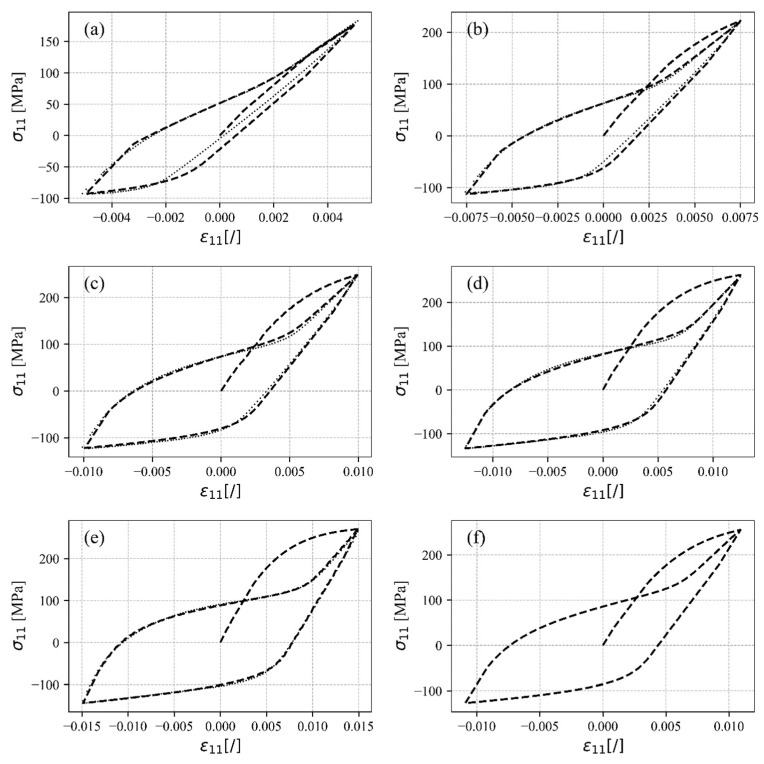
Simulated stress–strain responses under uniaxial tensile–compressive loading (dashed lines) compared to the experimental data (dotted lines) for (**a**) $\varepsilon_{a}\;$= ±0.5%, (**b**) $\varepsilon_{a}\;$= ±0.75%, (**c**) $\varepsilon_{a}\;$= ±1.0%, (**d**) $\varepsilon_{a}\;$= ±1.25%, (**e**) $\varepsilon_{a}$= ±1.5%, and (**f**) $\varepsilon_{a}$= ±1.1%.

After completing the calibration of the material parameters, the proposed constitutive model should be tested and validated against experimental observations. First, the validation of the uniaxial tensile–compressive loading is presented. Figure 7 shows the simulated results compared to the experimental data. It can be clearly seen that the proposed constitutive model can predict the stress–strain response very well, which is expected since the model was calibrated using these data. Figure 7f illustrates a simulated stress–strain response for a strain amplitude of $\varepsilon_{a}=\pm 1.1\%$, which falls between the experimental data points. This particular strain amplitude was not included in the experimental dataset. Despite this, the algorithm successfully simulates the response for this amplitude, demonstrating its capability of extrapolating accurately beyond the range of the experimental data. However, a limitation of this approach is that it cannot ensure a precise and accurate prediction of the response for strains exceeding the maximum or falling below the minimum values of the strain amplitudes of the experimental data.

**Figure 8:**
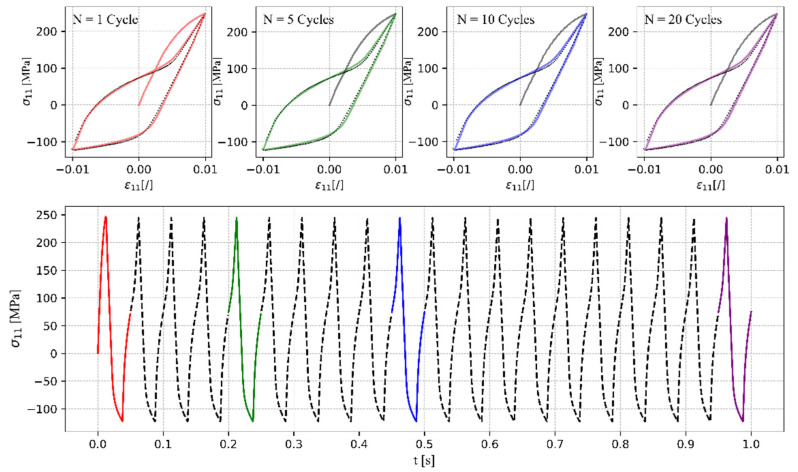
Simulated stress–strain responses to uniaxial tensile–compressive loading with a strain amplitude $\varepsilon_{a}=\pm 1.0\%$ for different numbers of simulated loading cycles using hardening parameters update algorithm.

Next, the consistency and the ability to perform closed hysteresis loops were tested for the case of uniaxial tensile–compressive loading with a strain amplitude $\varepsilon_{a}=\pm 1\%$. As shown in Figure 8, the response is consistent with the load and the number of cycles applied. The algorithm proposed in this study to update the hardening parameters ensures that non-drifting closed hysteresis loops can be simulated. On the contrary, a drift of the reversal points would be observed, which should not occur when simulating a stable cyclic response.

**Figure 9:**
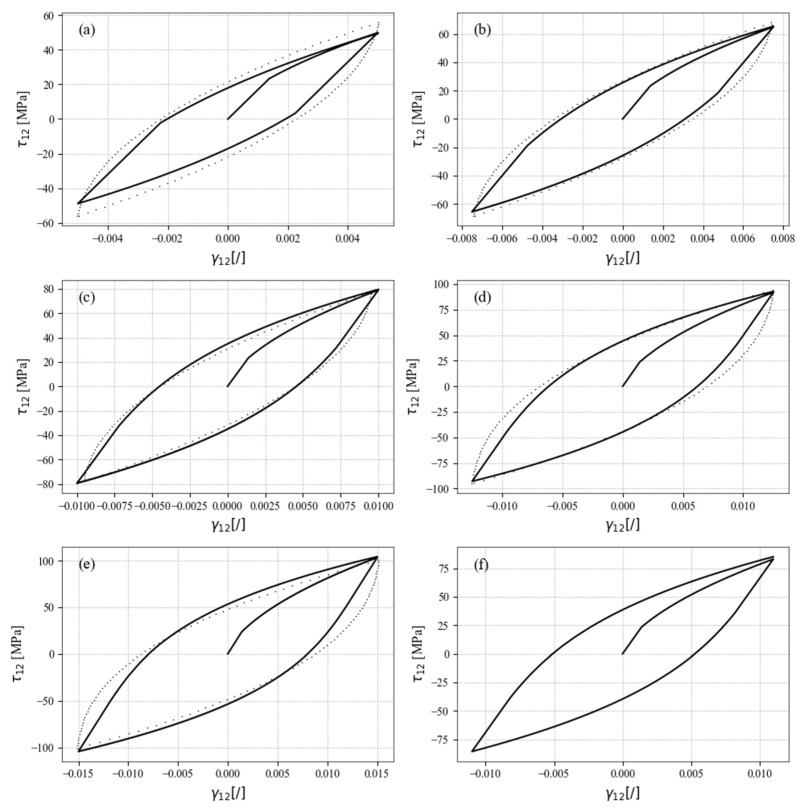
Simulated stress–strain responses to shear loading (solid lines) with respect to the experimental data (dotted lines) for (**a**) $\gamma_{a}\;$= ±0.5%, (**b**) $\gamma_{a}\;$= ±0.75%, (**c**) $\gamma_{a}\;$= ±1.0%, (**d**) $\gamma_{a}\;$= ±1.25%, (**e**) $\gamma_{a}\;$= ±1.5%, and (**f**) $\gamma_{a}\;$= ±1.1%.

The same validation study was then performed for pure shear loading. The calibrated constitutive model was compared to the experimental data for different amplitudes of shear stress, as shown in Figure 9. Again, the constitutive model simulated the responses correctly. Since the hardening is symmetrically defined and does not depend on the direction of the shear stress, the algorithm for updating the hardening parameters is not required, since the hysteresis loops are already closed and also do not depend on the number of cycles.

Figure 9 shows that the magnitude of the yield stress plays an important role in the simulations. It is visible here for the simulation of pure shear loads as a noticeable, non-continuous transition from the elastic to the plastic part of the constitutive model in comparison to the experimental data (dotted lines). This can be avoided by lowering the yield stress, but then the missing elastic part must be replaced by the correspondingly prescribed plastic hardening. However, the validity of the simulated response can become compromised. As shown, the proposed constitutive model can simulate pure uniaxial tensile–compressive and pure shear loading. Likewise, the static part of the stabilised response is also included in the simulations, as it is crucial for the comprehensive ability of the stress–strain response simulations. Figure 9f presents a shear strain amplitude that falls between the experimental data points. This demonstrates the model’s capability to interpolate and simulate responses accurately for strain amplitudes not explicitly tested in the experimental dataset. However, a limitation of this approach is that it may not reliably predict responses for shear strain amplitudes beyond the maximum or below the minimum experimental data points for which the model was calibrated.

### 4.3. Multiaxial Proportional and Non-Proportional Loading

**Figure 10:**
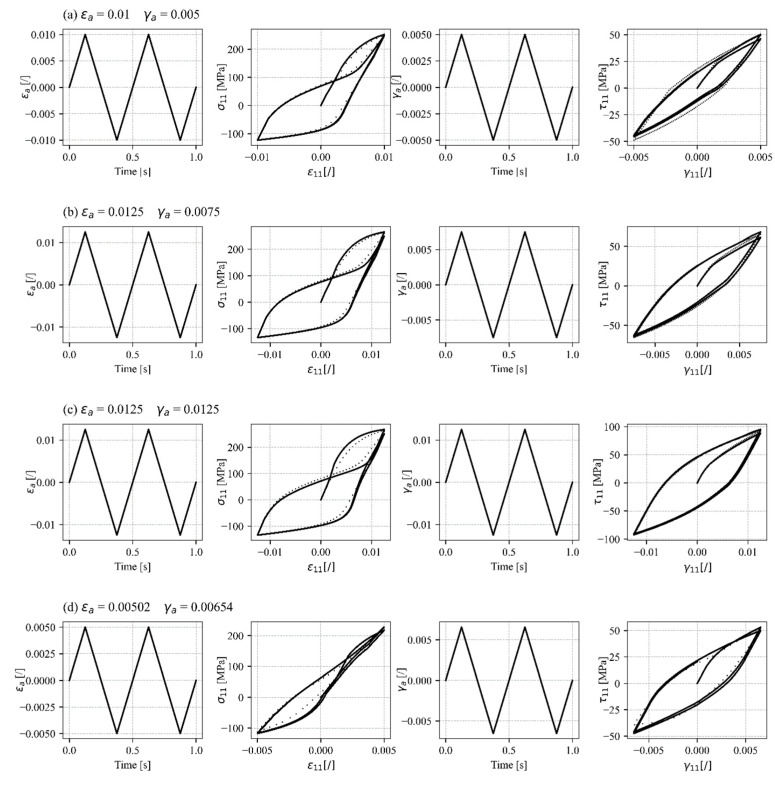
Simulated stabilised stress–strain responses under in-phase proportional loading compared to pure uniaxial tension–compression and pure shear loading (solid lines) (**a**) $\varepsilon_{a}$ = ±1%, $\gamma_{a}\;$ = ±0.5%, (**b**) $\varepsilon_{a}$ = ±1.25%, $\gamma_{a\;}$ = ±0.75%, and (**c**) $\varepsilon_{a}$ = ±1.25%, $\gamma_{a\;}$ =±1.25%, and against the experimental data (dotted lines) (**d**) $\varepsilon_{a}$ = ±0.502%, $\gamma_{a}\;$ = ±0.654%.

Proportional loading was used for a further validation of the proposed constitutive model. The simulated response is compared to the uniaxial tensile–compressive and shear response previously presented in this study, as there are currently no experimental data on proportional or non-proportional multiaxial loading of AZ31 sheet metal for these material parameters. Nevertheless, the proposed constitutive model is able to simulate the stabilised axial and shear stress–strain curves of proportional in-phase loading (Figure 10a–c). The sigmoidal stress–strain response can be clearly seen, as well as the asymmetric behaviour in tension and compression under uniaxial plastic deformation. The asymmetric shear behaviour appears in this case, which is a consequence of the twinning and untwinning saturation occurrence under normal tensile–compressive loading. All of the above phenomena were observed by Albinmousa when performing multiaxial experiments on the wrought magnesium alloy AZ31 under uniaxial tensile–compressive and torsional loading [86]. Figure 10d therefore shows the comparison between the simulated response and experimental data for the wrought magnesium alloy AZ31 [86] with modified hardening parameters. These parameters are again situated between the experimental data points for which the model was calibrated, demonstrating the model’s capability to interpolate and accurately simulate responses within this range.

The ability to simulate proportional and non-proportional multiaxial loads is very important, as this type of loading is most similar to loads in the real environment. The proposed constitutive model can therefore be applicable to simulations of the response under proportional loading. It should be noted, however, that the material parameters were calibrated on experimental results for AZ31 sheet metal under both uniaxial tensile–compressive and biaxial shear loading. In the future, multiaxial experiments should be performed on AZ31 sheets with uniaxial loading and in-plane shear loading to obtain a direct comparison between the simulation and the experiments.

## 5. Conclusions

The enhanced cyclic plasticity model enables simulations of AZ31 sheet metal under low-cycle fatigue conditions to observe experimentally consistent stress responses for the given strain load history. This has been achieved by the suggested robust stress update algorithm. Moreover, the constitutive model guarantees the closure of hysteresis loops, which has been addressed by the new hardening parameter update algorithm using the hyperbolic tangent function to describe hardening. The hardening parameters are iteratively updated until the optimal conditions are met. Finally, the constitutive model has been thoroughly tested and validated on the uniaxial experimental data, leading to excellent matching results for AZ31 sheet metal. A multiaxial proportional validation has also been performed and has shown promising results. However, it is recognised that further enhancements could be beneficial to improving the performance in predicting behaviour under multiaxial loading conditions. The current simulated response to multiaxial loading was compared against uniaxial tensile–compressive and torsional experiments on wrought magnesium alloy AZ31 from the literature, rather than multiaxial loading experiments on sheet metal specimens involving uniaxial tensile–compressive and in-plane shear loading. Multiaxial loading experiments on sheet metal are necessary to complement the model’s capabilities. The enhancements made to the hardening parameter definitions in this research are crucial for achieving a more accurate simulation of the response for AZ31. It is important to note that this work represents an upgrade to the existing constitutive model rather than the development of an entirely new model. The next step in the research into the proposed constitutive model is the integration of memory rules and the introduction of variable loading amplitudes with nested load cycles. The proposed model can be implemented into finite element analysis in the future.

## Figures and Tables

**Table 1:** Hardening parameters of the proposed constitutive model for stabilised cyclic response of AZ31 magnesium alloy sheet metal.

Dynamic Parameters	Value	Static Parameters	Value
$h_{U}$	645,057 MPa	$A_{11}$	$1.453\cdot 10^{7}$
$H_{0U}$	2000 MPa	$\sigma_{0,11}$	34.06 MPa
$h_{L1}$	−1,001,644 MPa	$B_{11}$	−0.9706
$d_{L1\;}$	70 MPa	$C_{11}$	−70,280 MPa
$h_{L2}$	55,000 MPa	$A_{12}$	$7.3\cdot 10^{5}$
$d_{L2\;}$	20 MPa	$\sigma_{0,12}$	5.12 MPa
$n_{s}^{\prime }$	0.3024	$B_{12}$	−0.99
$K_{s}^{\prime }$	379.42 MPa		

## Data Availability

The original contributions presented in the study are included in the article, further inquiries can be directed to the corresponding author.
